# Fast on-rates of chimeric antigen receptors enhance the sensitivity to peptide MHC *via* antigen rebinding

**DOI:** 10.1016/j.jbc.2024.107651

**Published:** 2024-08-08

**Authors:** Hiroyuki Hiratsuka, Yasushi Akahori, Shingo Maeta, Yuriko Egashira, Hiroshi Shiku

**Affiliations:** 1 Department of Personalized Cancer Immunotherapy, Graduate School of Medicine, Mie University, Tsu, Mie, Japan; 2 Bio-Diagnostic Reagent Technology Center, Sysmex Corporation, Kobe, Hyogo, Japan; 3 Center for Comprehensive Cancer Immunotherapy, Mie University, Tsu, Mie, Japan

**Keywords:** cancer, immunotherapy, chimeric antigen receptor, major histocompatibility complex (MHC), binding affinity, T cell, cell engineering, receptor, peptides

## Abstract

Chimeric antigen receptor (CAR) is a synthetic receptor that induces T cell-mediated lysis of abnormal cells. As cancer driver proteins are present at low levels on the cell surface, they can cause weak CAR reactivity, resulting in antigen sensitivity defects and consequently limited therapeutic efficacy. Although affinity maturation enhances the efficacy of CAR-T cell therapy, it causes off-target cross-reactions resulting in adverse effects. Preferentially expressed antigen in melanoma (PRAME) is an intracellular oncoprotein that is overexpressed in various tumors and restricted in normal tissues, except the testis. Therefore, PRAME could be an ideal target for cancer immunotherapy. In this study, we developed an experimental CAR system comprising six single-chain variable fragments that specifically recognizes the PRAME_p301_/HLA-A∗24:02 complex. Cell-mediated cytotoxicity was demonstrated using a panel of CARs with a wide range of affinities (*K*_D_ = 10^−10^–10^−7^ M) and affinity modulation. CAR-T cells with fast on-rates enhance antigen sensitivity by accelerating the killing rates of these cells. Alanine scanning data demonstrated the potential of genetically engineered CARs to reduce the risk of cross-reactivity, even among CARs with high affinities. Given the correlation between on-rates and dwell time that occurs in rebinding and cell-mediated cytotoxicity, it is proposed that CAR-binding characteristics, including on-rate, play a pivotal role in the lytic capacity of peptide-major histocompatibility complex-targeting CAR-T cells, thus facilitating the development of strategies whereby genetically engineered CARs target intracellular antigens in cancer cells to lyse the cells.

Chimeric antigen receptors (CARs) are synthetic receptors comprising an extracellular antigen recognition domain, a single-chain variable fragment (scFv), and intracellular signaling domains derived from the T-cell receptor (TCR) complex, and its costimulatory molecule. Over the past decade, CAR-engineered T cells have become a well-established treatment option for certain hematologic malignancies (1). However, the transmembrane proteins targeted by CARs are also expressed in healthy tissues, and cell-surface antigens expressed only in tumor cells are limited.

Cancer-specific antigens, such as cancer driver proteins, are abundant intracellularly. They are degraded by the intracellular ubiquitin–proteasome system and present in low concentrations as peptides on the cell surface by major histocompatibility complex (MHC) class I molecules, genetically encoded by the human leukocyte antigen (HLA)-A gene. The number of cognate peptide–MHC complexes (pMHCs) is 50 to 100 per cell, which is considerably lower than the number of overexpressed membrane proteins, for which approximately 3000 antigens per cell is the therapeutic target antigen density threshold for conventional CARs (2, 3). Furthermore, CARs have approximately 100-fold lower antigen sensitivity than TCRs. Antigen sensitivity defects in CAR-T cells limit the expansion of cancer-specific target antigens in the cell, such as pMHCs, thus limiting the efficacy of CAR-T cell therapy (4, 5).

Binding affinity, expressed as the dissociation constant (*K*_D_), is the equilibrium dissociation rate constant of the binding strength of a receptor molecule to the ligand and is defined as the ratio of the dissociation rate (off-rate) to the association rate (on-rate). In the case of TCRs that recognize pMHC, there is an affinity range (approximately *K*_D_ = 1–100 μM) for an optimal T cell response after stimulation of TCR-bearing T cells (6, 7). Focusing on binding parameters, such as the on-rate of binding affinity, fast on-rates allow short-dwell time ligands to activate T cells by gaining dwell time due to the increased frequency and duration of TCR rebinding to ligands (8). For CARs targeting pMHC, either TCR-like binding affinity, low affinity, or both are required (9, 10); however, even high-affinity binders with *K*_D_ ≤ 10^−8^ M can induce efficient CAR-T cell activation (11, 12, 13, 14). In CAR/pMHC interactions, binding affinity is a critical factor; however, it remains unclear how binding parameters, including on-rate, which constitute binding affinity, affect the antigen sensitivity of T cells *via* synthetic receptors.

In the current study, to understand the interplay between the tumor-killing activity of CAR-T cells and binding parameters to pMHC, we tested the killing efficacy of a panel of CAR-T cells against preferentially expressed antigen in melanoma (PRAME), which is the testis cancer antigen presented by HLA-A∗24:02 (15). PRAME is an intracellular oncoprotein that plays a pivotal role in suppressing proliferation arrest to prevent retinoic acid from binding to the retinoic acid receptor (16). It is overexpressed in various tumors such as melanoma and acute myeloid leukemia (15). PRAME overexpression in normal tissues is restricted to the testes (17). The amino acid residues 301 to 309 (LYVDSLFFL) of the PRAME protein were established as an epitope displayed by HLA-A∗24:02 (15). HLA-A∗24:02 is the most common type of HLA-A allele in Asian populations; as this allele contains the PRAME epitope, it is a potential target for CARs. We established an experimental system with six PRAME-specific scFvs, including two WTs (WT98 and WT163) isolated from the M13 phage display library and four affinity-enhanced mutants generated from WT98 using affinity engineering techniques for on-rate modulation (18, 19). The scFvs covered the intrinsic affinity range of the antibody (*K*_D_ = 10^−10^–10^−7^ M) (20, 21). This study was designed to clarify whether improved binding parameters in terms of faster on-rate enable the CAR-T cells to effectively identify tumors, especially low-antigen density tumors, providing rapid killing rates. Our study elucidates how binding parameters, including the on-rate of binding affinity, play a pivotal role in enhancing the lytic capacity of CAR-T cells to target intracellular oncoproteins and provides a strategy whereby genetically engineered CARs target intracellular antigens in cancer cells.

## Results

### Panel of PRAME_p301_-specific CAR-T cells induced antigen-specific degranulation against HLA-A∗24:02-transduced T2 cells

**Figure 1:**
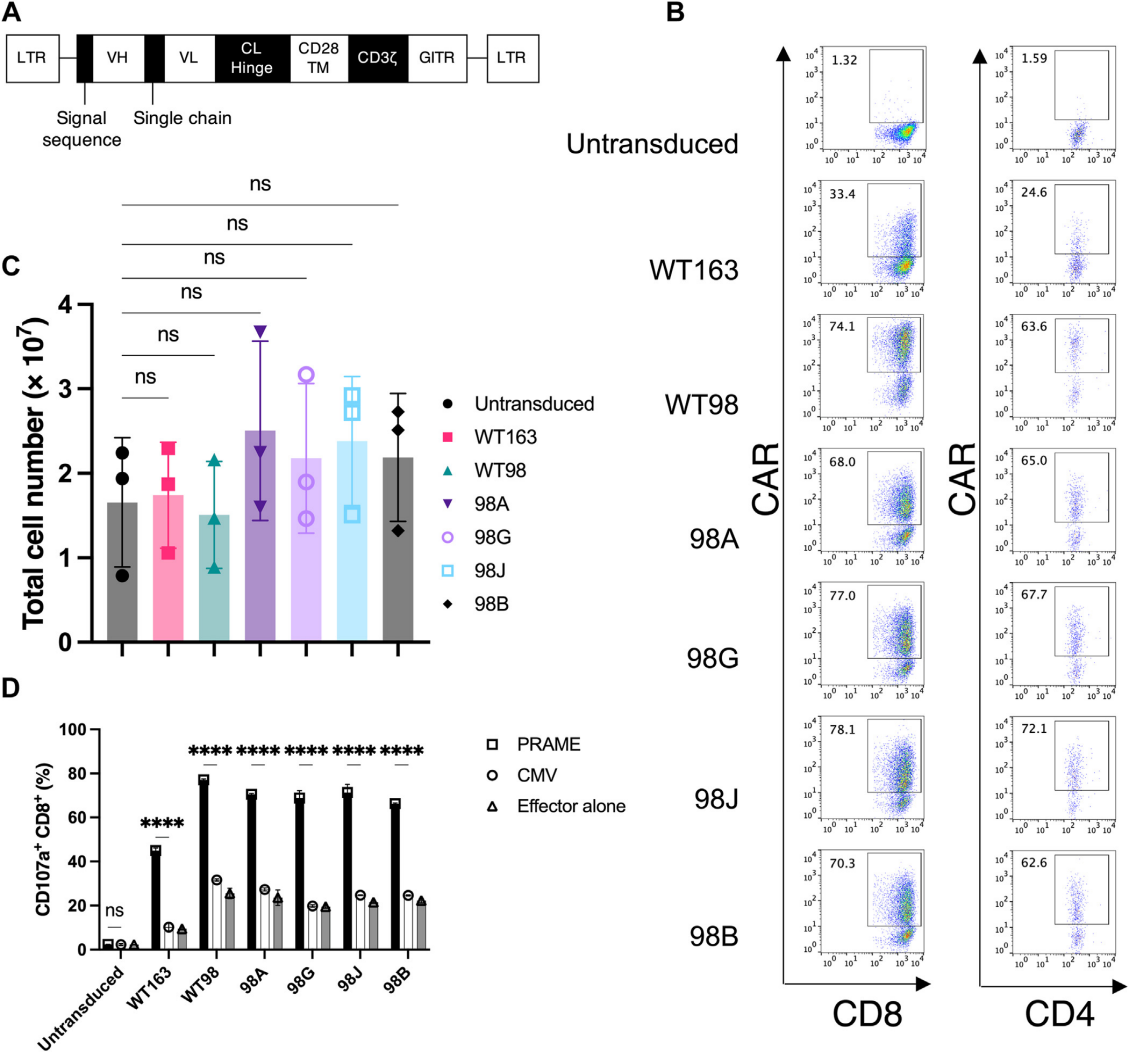
**Characterization of PRAME-specific CARs.***A*, schematic representation of the PRAME CAR construct containing the germline signal sequence. *B*, dot plots showing anti-lambda light chain staining for CAR expression in untransduced cells as background staining and WT163, WT98, 98A, 98G, 98J, and 98B CARs by flow cytometry. The *left* column of the dot plots shows CD8^+^ CAR^+^, whereas the *right* column shows CD4^+^ CAR^+^ in the representative data. *C*, expansion of PBMCs, including T cells, transduced with the six CARs. Data are presented as mean ± SDs of three independent experiments. *D*, PRAME_p301_/HLA-A∗24:02-dependent degranulation of CD8^+^ T cells. CAR-T cells were cocultured with T2A24 cells pulsed with 10 μM PRAME_p301_ or CMV_pp65_ peptides for 5 h, and then degranulated CD107a was measured by flow cytometry. Data are representative of two independent experiments and are shown as means ± SDs of duplicates. *C* and *D*, ∗∗∗∗*p* < 0.0001; ns, no significance was observed using two-way ANOVA with Tukey’s test. CAR, chimeric antigen receptor; CMV, cytomegalovirus; HLA, human leukocyte antigen; PBMC, peripheral blood mononuclear cell; PRAME, preferentially expressed antigen in melanoma.

We identified clones of WT98 and WT163 PRAME_p301_-specific scFvs using a phage display library (Table S1 and Fig. S1). To achieve different binding affinities for scFvs, four mutants were generated in the WT98 clone using affinity engineering (18, 19). The affinities of the scFvs were measured using surface plasmon resonance (SPR) (Table 1 and Fig. S2). The selected affinity range was approximately three logarithmic orders of magnitude, in accordance with previous studies on the optimal binding affinity for efficient responses of CAR-T cells (22, 23, 24). To investigate the antigen-specific activity of CAR-T cells, scFv-containing CAR-T cells were generated using a retroviral vector as previously described (25). The CAR constructs include the scFv domain, hinge domain of the constant region of the lambda light chain, intracellular signaling domain of CD3ζ, and glucocorticoid-induced tumor necrosis factor receptor family-related protein as well as a CD28 transmembrane domain (Fig. 1*A*). The CAR-T cells showed comparable cell-surface CAR expression in both CD4^+^ and CD8^+^ T cells (Fig. 1*B*). To evaluate the expansion of transduced human peripheral blood mononuclear cells (PBMCs), they were cultured under the same conditions and counted on day 7 postviral transduction. All transduced PBMCs proliferated with similar expansion efficiencies. Untransduced cells were used as controls (Fig. 1*C*). Next, to confirm the effector function of CAR-T cells, the expression of CD107a, a marker of CAR-T cell degranulation, was evaluated in T2 cells transduced with HLA-A∗24:02 (T2A24) and pulsed with the PRAME_p301_ peptide using flow cytometry (Fig. 1*D*). These experiments showed that the CAR-T cells produced CD107a. As expected, there was no response to T2A24 cells pulsed with cytomegalovirus (CMV)_pp65_ as a control peptide.

**Table 1:** Biophysical properties of antibodies binding to PRAME_p301_/HLA-A∗24:02

Clone	On-rate (10^5^/Ms) (±SE)	Off-rate (10^-2^/s) (±SE)	*K*_D_ (nM)	*Χ*^2^	U-value	Binding affinity
WT163	1.4 (±0.2)	1.53 (±0.2)	108	101	9	+
WT98	7.1 (±0.009)	1.33 (±0.01)	22	0.038	-	++
98A	93 (±0.3)	2.48 (±0.02)	2.7	0.18	-	+++
98G	160 (±0.03)	1.78 (±0.0003)	1.2	0.083	-	+++
98J	190 (±2.3)	0.94 (±0.01)	0.50	0.17	-	++++
98B	100 (±0.04)	0.26 (±0.001)	0.26	4.24	-	++++

Dissociation constant (K_D_), *Χ*^2^, and U-value have been provided for fitting models.

SE, standard error.

### Affinity-tuned CARs in terms of fast on-rates showed rapid killing rates against endogenously PRAME-expressing tumor cells

**Figure 2:**
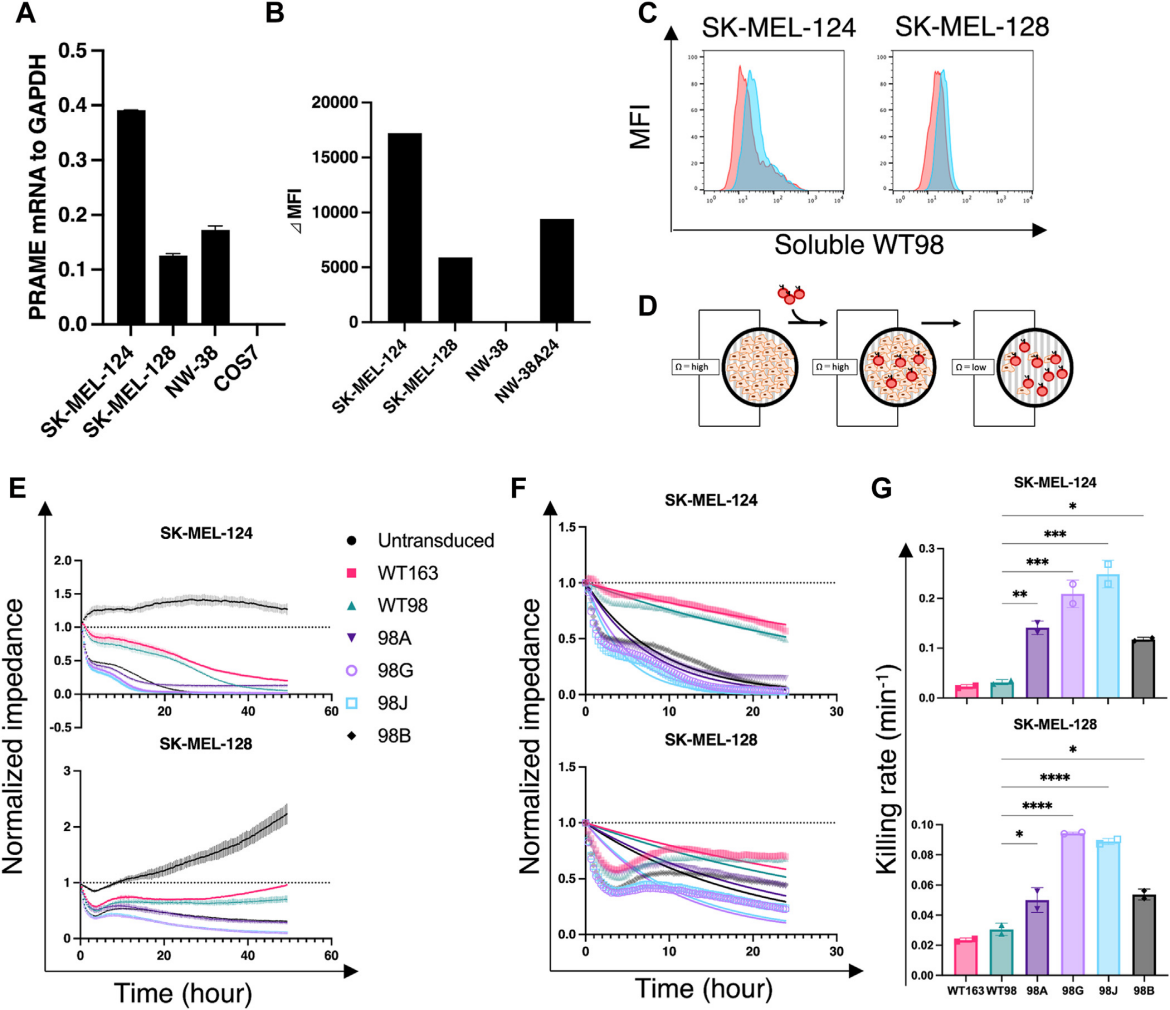
**CAR-T cells induce functional activity in endogenous PRAME-expressing tumor cells.***A*, PRAME mRNA expression was determined using qPCR. The COS-7 cell line was used as a negative control. *B*, HLA-A∗24:02 cell surface expression was detected using flow cytometry. Cells stained with Alexa488 alone indicate the background. *C*, the indicated cell lines were stained with or without soluble WT98 along with the secondary antibody, and then with the tertiary antibody conjugated with PE. *D*, schematic representation of *in vitro* real-time killing system. *E*, the tumor-killing capacity of CAR-T cells was measured by the continuous decrease in impedance of the xCELLigence system against SK-MEL-124 and SK-MEL-128 cell lines at an E:T ratio of 5:1. *F*, killing rates were calculated as rate constants using a one-phase exponential decay curve fit. Data are representative of the duplicates. *G*, killing rates of CAR-T cells against the SK-MEL-124 and SK-MEL-128 cell lines. *A*, *E*, and *G*, data represent the mean ± SDs of the duplicates. *E* and *G*, data are representative of two independent experiments. *G*, ∗∗∗∗*p* < 0.0001, ∗∗∗*p* < 0.001, ∗∗*p* < 0.01, ∗*p* < 0.05; ns, no significance observed using one-way ANOVA with Tukey’s test. CAR, chimeric antigen receptor; HLA, human leukocyte antigen; PRAME, preferentially expressed antigen in melanoma; qPCR, quantitative PCR.

It is crucial to determine whether CAR-T cells respond to the endogenous PRAME peptide displayed by HLA-A∗24:02. Therefore, we confirmed the expression of PRAME and HLA-A∗24:02 in the melanoma cell lines, SK-MEL-124, SK-MEL-128, and NW-38, using the African green monkey kidney fibroblast-like cell line, COS-7, as a PRAME-negative control and HLA-A∗24:02-transduced NW-38 (NW-38A24) as an HLA-A∗24:02-positive control (Fig. 2, *A* and *B*). When comparing PRAME and HLA-A∗24:02 expression in SK-MEL-124 and SK-MEL-128 cells, SK-MEL-124 expressed PRAME and HLA-A∗24:02 at high levels (PRAME/A24^hi^), and SK-MEL-128 expressed PRAME and HLA-A∗24:02 at low levels (PRAME/A24^low^). During the detection of cognate pMHCs using soluble WT98 (Fig. S3), the cognate pMHC level of SK-MEL-124 was higher than that of SK-MEL-128, consistent with PRAME mRNA and HLA-A∗24:02 expression (Fig. 2*C*). To directly investigate tumor cell death, we used a real-time cell analyzer, the xCELLigence system (Agilent) (Fig. 2*D*). This system monitors the viability of adherent cells in real time by measuring the impedance of unlabeled cells attached to electrodes in culture wells in a CO_2_ incubator for 2 to 3 days and is often used in studies on cancer metastasis and immune response (26, 27, 28). As the adherent cells die, their adhesion resistance weakens and the impedance decreases. This electric impedance-based assay has an advantage over the classical lysis assay because it is more sensitive and can track changes over time (29). At an effector-to-target ratio (E:T) of 5:1, CAR-T cells carrying six CARs lysed PRAME/A24^low^ and PRAME/A24^hi^ target cells (Fig. 2*E*). The killing capacity of CAR-T cells against NW-38A24 cells was also confirmed (Fig. S4). However, CAR-T cells did not lyse the NW-38 cells (Fig. S4*B*). For TCRs with a fast on-rate, T cells with ligands that exhibit short dwell times can induce efficient activation by rebinding (8). Therefore, we hypothesized that CARs with fast on-rates also contribute to efficient T cell activation *via* rebinding between CAR and pMHC. Therefore, we investigated the killing rate of CAR-T cells. Based on the first-order equation, killing rates can be used to measure the lysis of cytotoxic T cells under culture conditions in a dish (30), and to assess the killing capacity of T cells by fitting the rates to a one-phase exponential decay model (31). To calculate the killing rates of CAR-T cells from the normalized impedance of real-time tumor cell death, the data were used to generate a one-phase exponential decay equation using the GraphPad Prism 9 (GraphPad Software, https://www.graphpad.com) Equation 1.

(1) $$Y=\left(Y0-Plateau\right)e^{-kX}+Plateau$$

To measure the killing rates of CARs, we calculated the impedance change over 24 h (Fig. 2*F*). The killing rates of T cells containing six CARs against the SK-MEL-124 cell line increased with binding affinity, except for the variant 98B. The faster on-rate variants 98A, 98G, and 98J (which have off-rates >1 × 10^−2^/s) exhibited 4- to 8-fold increase in killing rates compared with WT98; however, variant 98B with fast on-rates and slow off-rates (<1 × 10^−2^/s) exhibited poorer killing rates (Fig. 2*G*). This trend in CAR killing rates was also observed in SK-MEL-128 cells with low antigen density. CAR-killing rates against lower antigen density indicated a reduction in killing rates. Moreover, to investigate the short-term CAR-T cell response, a degranulation assay was performed after stimulating CAR-T cells for 1 h; CARs with fast on-rates, *i.e.*, 98A, 98G, and 98J, induced higher degranulation than WT98 against T2A24 cells pulsed with 10 nM PRAME peptide (Fig. S5). These results support the rapid killing rates with fast on-rates and off-rates (>1 × 10^−2^/s between these variants). To further investigate the killing capacity of the CAR panel, we examined whether a lower E:T ratio than that used in Figure 2*E* would reveal differences in the tumor-killing capacity of CAR-T cells with different binding affinities. CAR-T cells with fast on-rates and off-rates eliminated PRAME/A24^hi^ target cells (Figs. S6*A* and S7*A*), even at lower E:T ratios of 0.5:1 (Figs. S6*B* and S7*B*) and 0.15:1 (Figs. S6*C* and S7*C*) compared to WT98 CAR-T cells after 50 h of coculture. High-affinity CAR-T cells showed potent killing capacity when exposed to PRAME/A24^low^ target cells at E:T = 0.5:1, whereas CAR-T cells with fast on-rates and slow off-rates showed decreased killing capacity compared to CAR-T cells with fast on-rates and off-rates against WT98 (Figs. S6*B* and S7*B*). These results support the idea that higher-affinity CARs with fast on-rates and off-rates are more capable of lysing endogenous PRAME-expressing tumor cells than lower-affinity CARs. Kinetic measurements at effector ratios of 1.5–0.5 are not shown because the normalized impedance curves did not reach a sufficient plateau phase in CAR-T cell target lysis except against the SK-MEL-124 cell line at E:T = 1.5:1.

### Alanine scanning data demonstrate the antigen-recognition properties of CAR-T cells

**Figure 3:**
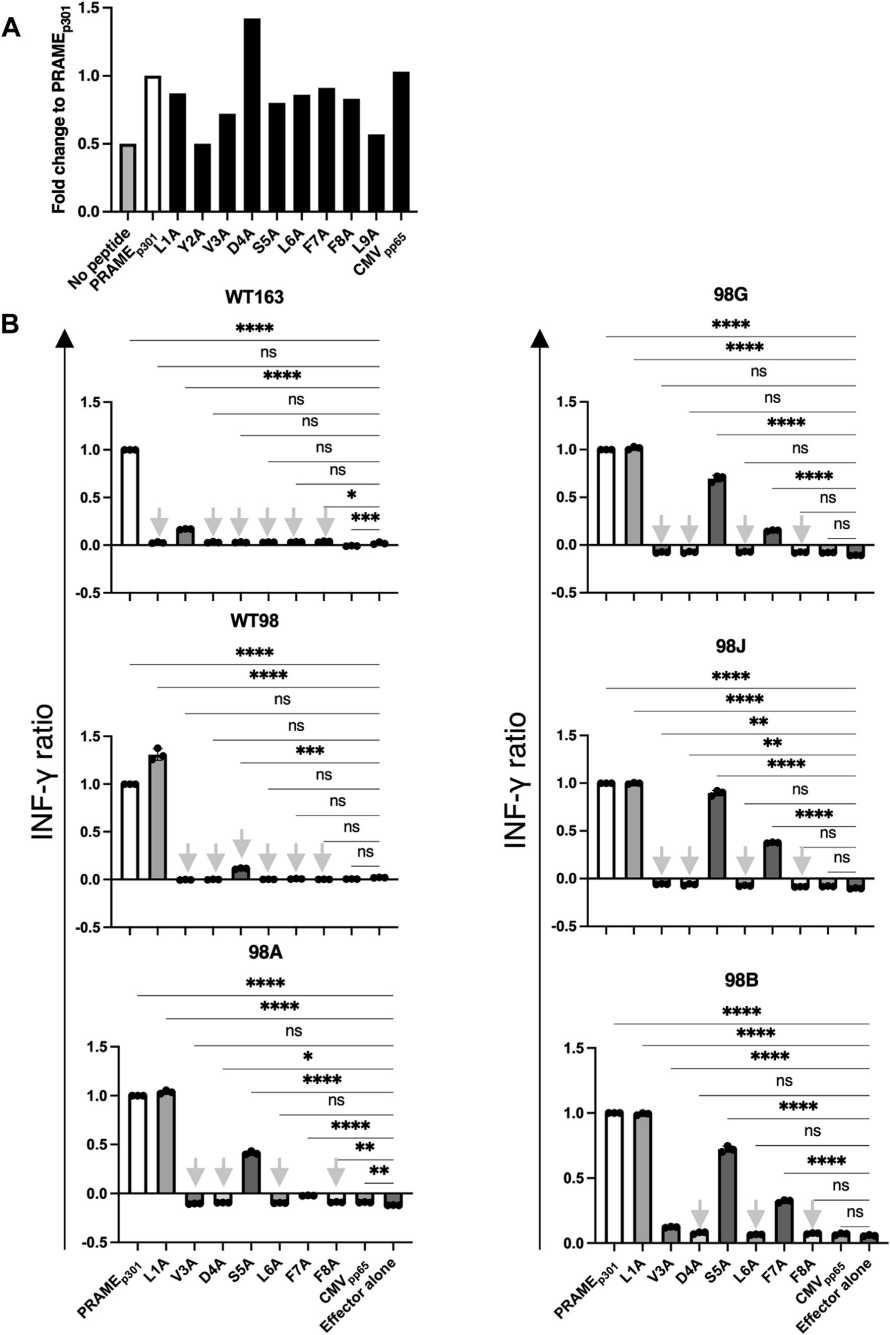
**Alternative specificity for CAR-T cells containing six unique scFvs.***A*, relative HLA-A∗24:02 binding stability of each peptide to determine the amino acid residues that bind HLA-A∗24:02. T2A24 cells were loaded with peptides (50 μM) overnight, and HLA-A∗24:02 expression on the cell surface was measured using flow cytometry and compared with that of unloaded T2A24. *B*, alanine scanning of LYVDSLFFL identified significant peptide residues for recognition by CAR-T cells that carry six unique scFvs. CAR-T cells were incubated for 24 h with T2A24 cells pulsed with each peptide (300 nM) and the ratio of IFN-γ to PRAME_p301_ was measured in the culture supernatants. Based on the significance of the effector alone and the positive control PRAME_p301_, amino acids with *gray arrows* were identified as those that abolished CAR binding. Data are shown as mean ± SDs of triplicate experiments. Data are representative of two independent experiments. *B*, ∗∗∗∗*p* < 0.0001, ∗∗∗*p* < 0.001, ∗∗*p* < 0.01, ∗*p* < 0.05; ns, no significance observed using one-way ANOVA with Tukey’s test. CAR, chimeric antigen receptor; HLA, human leukocyte antigen; IFN-γ, interferon gamma; PRAME, preferentially expressed antigen in melanoma; scFV, single-chain variable fragment.

Binding to cognate pMHC increases the risk of cross-reactivity with off-target antigens for T cells bearing high-affinity receptors (32), including high-affinity CARs. Therefore, to evaluate the antigen specificity and risk of cross-reactivity with other peptides, we performed alanine scanning to identify the significant amino acid residues that facilitate the binding of the CARs to PRAME/HLA-A∗24:02 with peptides that substituted each position of the PRAME peptide with alanine (33). We prepared synthetic peptides (Table S2) and evaluated whether alanine altered the stability of the peptides to bind to HLA-A∗24:02 using an HLA stabilization assay (Fig. 3*A*). The results showed that substitutions at positions 2 and 9 were associated with a decrease in HLA-binding capacity, consistent with these positions being the primary anchors of the canonical HLA-A∗24:02. Next, interferon gamma (IFN-γ) production by CAR-T cells co-cultured with T2A24 cells was evaluated by a peptide panel to determine which amino acids of peptides on the HLA-A∗24:02 were involved in CAR-T cell recognition. Substituted amino acids that decrease IFN-γ production are important for CAR-mediated antigen recognition. Alanine scanning revealed that WT163 recognized amino acids at positions 1, 4, 5, 6, 7, and 8, and WT98 recognized amino acids at positions 3, 4, 5, 6, 7, and 8. The high-affinity variants 98A, 98G, and 98J interacted with amino acids at positions 3, 4, 6, and 8. Variant 98B recognized amino acids at positions 4, 6, and 8. These statistics were obtained by comparing IFN-γ production by the effectors alone. Amino acids that showed high significance (*p* < 0.0001), equal to that of the PRAME_p301_ peptide, were considered to be strongly involved in CAR-mediated cross-recognition, because the peptide was recognized even when replaced by alanine (Fig. 3*B*). Therefore, the amino acids marked by gray arrows in Figure 3*B* are critical for CAR-mediated recognition of the PRAME_p301_ peptide. In addition, to investigate the risk of cross-reactivity, we searched for peptides that possess sequences similar to those derived from the human genome for “xYxDSLFFL,” “xYVDSLFFL,” “xYVDxLxFL,” and “xYxDxLxFL” using MOTIF Search (https://www.genome.jp/tools/motif/MOTIF2.html) and found only “xYxDxLxFL.” The peptides for “xYxDxLxFL” are listed in Table S3.

### Antigen sensitivity of CAR-T cells correlated with on-rates

**Figure 4:**
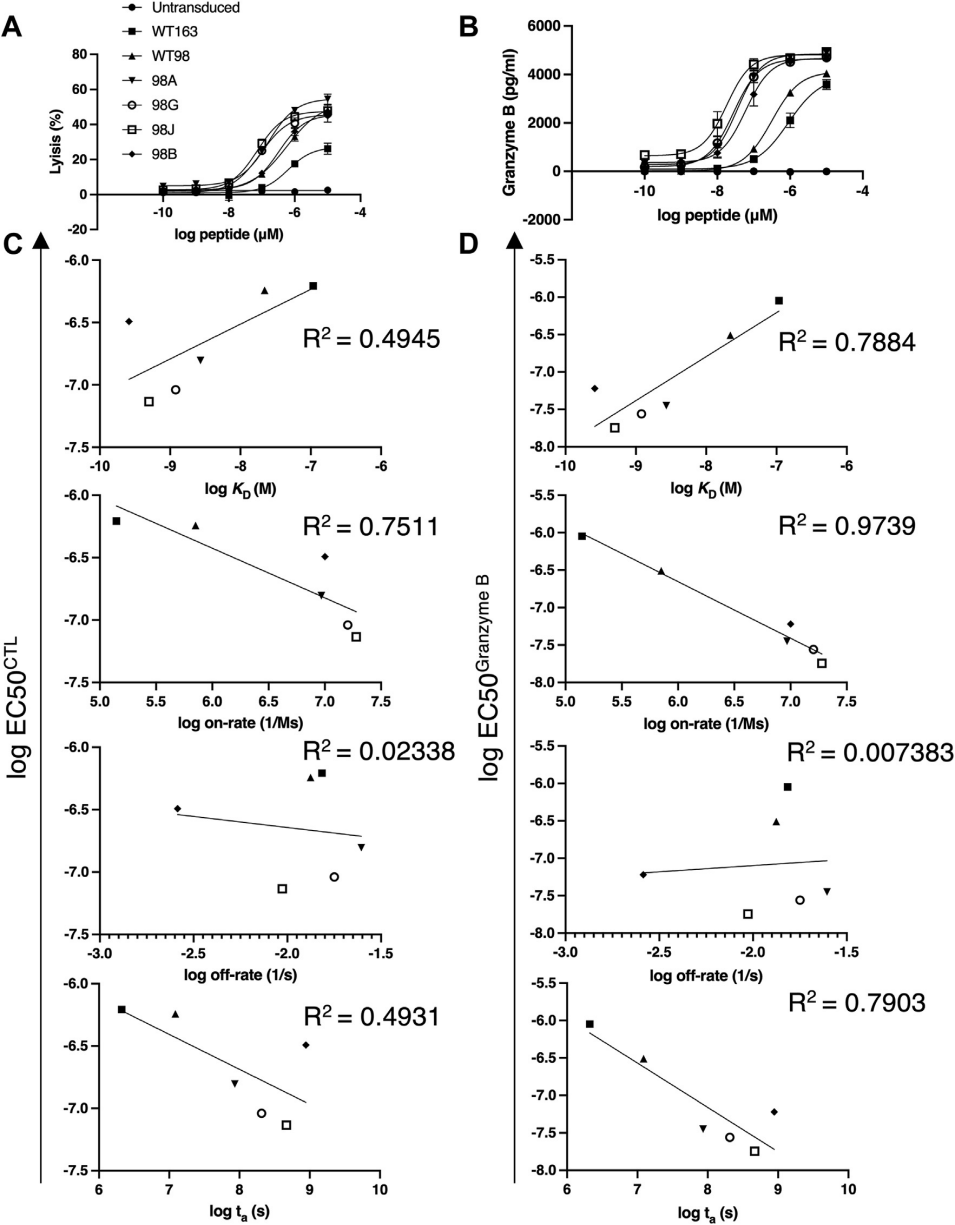
**Relationship between CAR-T cell ligand potency and antibody binding affinity.***A*, dose-response curve based on cytotoxicity induced by LDH release. Lysis% of CAR-T cells coincubated for 4 h with T2A24 cells pulsed with the indicated PRAME_p301_ peptide. *B*, dose-response curves of granzyme B secretion by CAR-T cells compared with T2A24 cells pulsed with the indicated PRAME_p301_ peptide. ELISA was used to measure the levels of secreted granzyme B. *C*, EC_50_ for lysis% from the LDH-based cytotoxicity assay plotted over the *K*_D_, on-rate, off-rate, and t_a_ in log-log-transformed values. *D*, EC_50_ of secreted granzyme B of CAR-T cells plotted over the *K*_D_, on-rate, off-rate, and t_a_ on log-log-transformed values. The *solid lines* represent the fit to the simple linear regression. Data represent two independent experiments and are presented as mean ± SDs of duplicates. CAR, chimeric antigen receptor; LDH, lactate dehydrogenase; PRAME, preferentially expressed antigen in melanoma.

To investigate the relationship between the antigen sensitivity of CAR-T cells and the binding parameters of scFvs as measured by SPR, we investigated EC_50_, which is the concentration that produces 50% of the maximal response in the lytic and secretory events of CAR-T cells. CAR-T cells were incubated with T2A24 cells treated with the indicated peptide doses, and their cytotoxicity and granzyme B secretion were evaluated (Fig. 4*A* and *B*). Generally, it is difficult to transform SPR-based on- and off-rates into on- and off-rates for ligands and receptors on the cell membranes (34, 35). Herein, based on the occupancy of the receptor/ligand (36), the on- and off-rates measured by SPR were converted into on- and off-rates on the membrane. We observed a strong correlation between cytotoxicity and the on-rate (Fig. 4*C*). Granzyme B production was also strongly correlated with the on-rate (Fig. 4*D*). However, the off-rate did not correlate with cytotoxicity or granzyme B production (Fig. 4, *C* and *D*). Furthermore, to investigate whether rebinding of CARs to the pMHC on the cells influences the total SPR-measured dwell time (t_a_) of rebinding that occurs at fast on-rates, t_a_ was calculated using Equation 2 in Govern’s model (8). Diffusion constants of a pMHC and a second-generation CAR were established at 0.04 μm^2^/s and 0.44 μm^2^/s, as typically measured in experiments on pMHCs (8, 37) and a CAR (38), respectively.

(2) ta=t12+[ln(2)2π(DCAR+DpMHC)]∙on−rateoff−rate

The parameter t_1/2_ was calculated by dividing ln (2) by the off-rate. *D*_*CAR*_ and *D*_*pMHC*_ are the diffusion constants of CAR and pMHC, respectively. Moreover, the EC_50_ values for cytotoxicity and granzyme B production were correlated with t_a_ and *K*_D_ (Fig. 4, *C* and *D*). This mathematical model depends on the *K*_D_ value, but the above results suggest that fast on-rates and off-rates allow CARs to rebind to cognate pMHC, which increases antigen sensitivity.

## Discussion

We used a CAR with six scFvs that recognize PRAME/HLA-A∗24:02 to demonstrate that adjusting parameters, including on-rates, which constitute the dissociation constant of CAR/pMHC, enhances antigen sensitivity. Increasing the on-rate leads to increased binding affinity. Depending on the technique used, affinity maturation increases the risk of off-target toxicity (39). However, the alanine scanning data obtained in this study showed that CAR interacts with three to six amino acid residues of the PRAME peptide. Moreover, simplified mutational analysis did not identify similar peptides in the human genome, except for variant 98B. Given that T cells need to bind 3 to 7 amino acids of peptides presented by the MHC through complementarity-determining regions in order to identify nonself antigens *via* the TCR (40, 41), it is suggested that scFv-based complementarity-determining regions also require 3 to 7 amino acids to bind peptide MHC. It is important to note that before preclinical studies can be initiated, a precise mutational analysis using X scanning with 20 amino acid substitutions for each amino acid of the PRAME_p301-309_ peptide may be necessary. Although six amino acids of the PRAME peptide are required for antigen recognition by WT98 CAR-T cells, soluble WT98 (which does not require an intervening cellular response) reacted with NW-38, which contains endogenous PRAME^+^/HLA-A∗02:01^+^ (Fig. S4*A*). Interestingly, the PRAME_p300-309_ peptide ALYVDSLFFL is presented by HLA-A∗02:01 (42). The recognition properties of the soluble WT98 in relation to the PRAME_p300-309_/HLA-A∗02:01 complex may differ from those based on CAR-T cells.

To maintain the antigen specificity of scFvs as chimeric immunoreceptors in T cells, a pMHC-restricted CAR requires a TCR-like affinity with *K*_D_ = 715 nM (9). However, CAR-T cells targeting intracellular proteins such as the cancer testis antigen NY-ESO-1, PRAME, and PHOX2B, a transcriptional regulator derived from neuroblastoma-dependent genes, have been shown to require an affinity of approximately 4 to 15 nM, rather than the TCR-like affinity range of *K*_D_ = 1 to 100 μM (11, 12, 43). The findings of this study demonstrate that the activation of pMHC-restricted CAR-T cells with fast on-rates and off-rates depends on the dwell time. Regarding the two variants 98A and 98B, which have similar on-rate values but different off-rate and *K*_D_ values, the CAR/pMHC interaction at a fast on-rate, with fast off-rates, led to more rapid killing than that at high *K*_D_ values caused by a slow off-rate. The killing rate of CAR-T cells was observed both in target cells with low and high antigen density. These results suggest that fast on-rate and off-rate are crucial for cellular responses, which directly affect the antigen sensitivity of CAR-T cells.

Although membrane proteins, such as CD19 and cognate pMHC, display a drastic difference in the number of cell-surface antigens, pMHC-restricted CARs containing CD28-based intracellular domains exhibit TCR-like nonmonotonic responses as a bell-shaped curve when plotted against different antigen densities (10). Moreover, phenotypic modeling of CAR-T cell activation only partially describes efficient CAR-T cell activation *via* kinetic proofreading (44). Consistent with previous studies, 98B CARs with *K*_D_ = 0.26 nM possess weak killing activity, suggesting the presence of a bell-shaped affinity threshold. However, the 98J CAR at *K*_D_ = 0.5 nM, which is similar to 98B CAR at *K*_D_ = 0.26 nM, showed increased killing activity. This result suggests that fast on-rate and off-rate increase CAR sensitivity. Furthermore, biophysical forces contribute to cell-cell interactions and may explain the efficient activation of CAR-T cells against pMHC. For example, single molecular measurements of the TCR and pMHC on T cells have shown that mechanical pulling forces play an important role in T-cell antigen recognition (45, 46, 47). In the case of CARs, transmembrane molecules such as CD19 ligands detect slip bonds in mechanical force measurements between CARs and antigens, whereas pMHC ligands detect catch bonds (48). Catch-bond formation contributes to cellular responses, such as fluidity and dynamics, rather than receptor-ligand binding properties, such as the on-rate constant (49), through a mechanism that efficiently activates exogenously transduced affinity-tuned TCRs on T cells targeting pMHC (50). If the activation of pMHC-targeting CAR-T cells is related to the catch-bond behavior as observed in T cells, insufficient mechanical force may cause CAR-T cells to form slip bonds. This hypothesis is supported by the fact that CAR-T cells containing other variants, such as 98I (mutation R63, R65, and R72), (*K*_D_ = 0.74 nM, on-rate = 150 × 10^5^/Ms, and off-rate = 1.14 × 10^−2^/s) and 98D (mutation R63, R70, and R72), (*K*_D_ = 2.3 nM, on-rate = 78 × 10^5^/Ms, and off-rate = 1.76 × 10^−2^/s) were poorly activated in T2A24 cells pulsed with the indicated PRAME_p301_ peptides and PRAME/HLA-A∗24:02^+^ tumor cell lines, despite their high affinity (data not shown). This scenario remains speculative and may be resolved by measuring the mechanical forces using the same cells.

Our study has several limitations. The optimal TCR-binding affinity required for efficient T cell activation differs between *in vivo* and *in vitro* experiments (51, 52). Therefore, there may be a difference in effective CAR-T cell activation *via* CAR/pMHC binding affinity between *in vivo* and *in vitro* experiments. Further *in vivo* experiments are required to confirm this. Moreover, there is a lack of understanding regarding the antigen sensitivity of on-rate-based affinity-tuned CARs compared to the TCR/CD3 complex-based chimeric receptors, such as TRuC, STAR, and HIT (53, 54, 55), and TCRs that recognize the PRAME/HLA-A∗24:02 complex (15). The contribution of accessory molecules and the CAR density must also be investigated further (5, 56, 57). When the TCR has an affinity of *K*_D_ ≤ 3 μM, the binding of CD8 to pMHC is not required. Therefore, it is possible that CARs with higher affinity than the TCRs (*K*_D_ ≤ 3 μM) also do not require binding to CD8 (58). In addition, there is a distinction between our CAR constructs and conventional CAR constructs. The latter includes the hinge domains CD8 or CD28 and the signaling domains CD28 or 4-1BB.

Nonetheless, this study highlights the importance of CAR-binding parameters, particularly the on-rate, in improving antigen sensitivity to pMHCs. These data indicate that synthetic receptors, including CARs that customize the intracellular signal domains, can target intracellular antigens, similar to naïve T cells that target intracellular antigens. This could dramatically expand the cancer-specific targets of synthetic receptors.

## Experimental procedures

### Cell lines

The following cell lines were used in this study: transporter associated with antigen processing (TAP)-deficient T2 cells transfected to express HLA-A∗24:02 (T2A24), SK-MEL-124 (PRAME^+^/A24^+^), SK-MEL-128 (PRAME^+^/A24^+^), NW-38 (PRAME^+^/A24^−^), NW-38 transfected to express HLA-A∗24:02 (NW-38A24), and COS-7 (PRAME^−^/A24^−^) cell lines. The TAP-deficient T2 (CEM × 721.174.T2) cell line, COS-7 cell line (an African green monkey kidney fibroblast-like cell line derived from the CV-1 monkey kidney cell line), and the other cell lines were provided by Dr Lloyd J. Old from the Memorial Sloan Kettering Cancer Center for the late Hiroshi Shiku. All cell lines were cultured in RPMI-1640 (Gibco, Thermo Fisher Scientific) supplemented with 10% fetal calf serum (Gibco), 20 mM Hepes (Gibco), 2 mM glutamine, 100 U/ml penicillin, and 100 μg/ml streptomycin.

### Isolation of scFvs specific to PRAME_p301-309_/HLA-A∗24:02 complex

We chose the PRAME_p301-309_/HLA-A∗24:02 complex as a target antigen for screening scFvs from an original human scFv M13 phage display library (7.5 × 10^11^ clones). To reduce the number of MHC-binding clones, biotinylated negative antigens from the HLA-A∗24:02 binding peptide_p30_ derived from cytomegalovirus pp65 were mixed with the human scFv phage library. Subsequently, phages that were bound to HLA-A∗24:02 as a complex with an irrelevant peptide were removed. Positive clones were screened using biotinylated PRAME_p301-309_/HLA-A∗24:02 complex-bound magnetic beads (Dynabeads MyOne, Invitrogen) at 4 °C for 1 h. The magnetic beads were trapped using a magnet-trapper and then washed with PBS and transfected into *Escherichia coli* DH12S (Invitrogen). The bacteria were cultured in 2 × YT (yeast extract tryptone) medium with 200 μg/ml ampicillin at 37 °C for 2 h and then spread on 2 × YT agarose plates containing a final concentration of 0.5% glucose and 200 μg/ml ampicillin (2 × YTAG media) at 37 °C for 15 h. After collecting 2 × YTAG media, the bacteria were infected with the VCSM 13 helper phage and cultured at 37 °C for 2 h. Kanamycin and IPTG were added at a final concentration of 30 μg/ml and 0.5 mM, respectively, and incubated at 28 °C for 20 h. After centrifuging the media at 7000*g* and 4 °C for 10 min using Himac CR21G (Hitachi Koki), the supernatant was harvested, concentrated *via* PEG precipitation, and suspended in PBS. Panning was performed over three cycles. The medium was added so that the scFv phage clones were expressed in the bacteria, and the supernatant containing scFvs was harvested. Positive phage clones were determined *via* ELISA against biotinylated recombinant PRAME_p301-309_/HLA-A∗24:02 complex in 1 μg of neutravidin-coated 96-well plates (Thermo Fisher Scientific). We selected 256 positive clones that bound to 1 μg of the PRAME_p301-309_/HLA-A∗24:02 complex/well in a 96-well plate using ELISA. Furthermore, to screen for clones recognizing low antigen density, we investigated 256 positive clones that bound to 0.5 μg of PRAME_p301-309_/HLA-A∗24:02 complex/well on the 96-well plate using ELISA and selected 40 positive clones. After sequence analysis of these clones, we identified 13 positive clones with different sequences. We investigated their inability to bind to irrelevant pMHCs and identified two positive clones that bound specifically to PRAME pMHC (Fig. S1).

### Purification of the scFvs

Soluble WT163 and WT98 scFvs fused with delta Protein A at the C terminus were inserted into pTZ19R vector and transfected into *E. coli* DH12S cells. The bacteria were cultured overnight at 30 °C by shaking in 400 ml of 2 × YT medium with 200 μg/ml ampicillin and 0.2 mM IPTG. The supernatant was harvested by centrifugation and mixed with an equal volume of water-saturated ammonium sulfate. After centrifugation, the pellet was suspended in PBS, and the supernatant was added to an IgG Sepharose column (GE HealthCare). The column was washed with 0.05% Tween PBS and PBS and then eluted with 0.2 M glycine–HCl (pH 2.5). The eluted solution was neutralized with 1 M Tris–HCl (pH 9.0). After dialysis with PBS, the sample solution was subjected to SDS-PAGE and Coomassie staining to check the size. Furthermore, to prepare WT98 and WT98 mutant antibodies for SPR measurement, human Fc-fused WT98 and mutant antibodies were expressed by transfecting the pcDNA 3.4 TOPO vector with the Expi293 expression system (Thermo Fisher Scientific), according to the manufacturer’s protocol. Antibodies were loaded onto a Protein A column (Cytiva) and eluted with arginine antibody elution buffer (Nacalai Tesque). Size-exclusion chromatography purification was conducted using Superdex 200 increase column (Cytiva). HBS-EP buffer (10 mM Hepes [pH 7.4], 150 mM NaCl, 3 mM EDTA, and 0.05% surfactant P20) was used as the running buffer for size-exclusion chromatography.

### Surface plasmon resonance

The binding kinetics and affinity of the WT163 scFv bound to the specific pMHC were measured *via* SPR using Biacore X100 (GE HealthCare). Biotinylated PRAME_301-309_/HLA-A∗24:02 complex was immobilized on a biotin capture sensor chip (GE HealthCare) at a final concentration of 50 nM using HBS-EP buffer. The titrated WT163 scFv protein was flowed at 30 ml/min in the flow cell at 25 °C. Moreover, to evaluate the kinetic parameters for WT98 and WT98 mutant antibodies, SPR experiments were performed using a Biacore 8K system (Cytiva). Ligand immobilization for the biotinylated PRAME_301-309_/HLA-A∗24:02 complex was conducted using a biotin capture sensor chip (Cytiva). The concentration was adjusted to 50 nM using HBS-EP buffer. WT98 and its mutants were injected over the immobilized antigen at a flow rate of 30 μl/min. The data were normalized by subtracting the response from that of the blank flow cell. Kinetic parameters were calculated using a bivalent-binding model.

### Production of MHC and human β-2-microgobulin inclusion body

HLA-A∗24:02 and human β-2-microglobulin proteins were produced in *E. coli* as inclusion bodies, using the following protocol. HLA-A∗24:02 or human β-2-microglobulin was cloned into the pET vector and transformed into BL21(DE3) RP *E. coli*. The bacteria were cultured in 1 ml LB medium containing 0.4% glucose, 50 μg/ml chloramphenicol, and 100 μg/ml ampicillin and then shaken at 250 rpm and 37 °C for 6 h. Then, 1 ml of culture medium was added to 100 ml LB medium containing 0.4% glucose, 50 μg/ml carbenicillin, and 100 μg/ml ampicillin and shaken at 37 °C overnight. Subsequently, 1/25 dilution of the culture (*A*_600 nm_ < 0.1) was shaken at 37 °C for 1.5 h (*A*_600 nm_ = −0.5). IPTG was added to the culture to reach a final concentration of 0.4 mM and the culture was shaken at 37 °C for 4 h. The bacterial culture was centrifuged at 5000*g* for 15 min at 4 °C. The bacterial pellet was resuspended in 10 ml of buffer A containing 50 mM Tris–HCl (pH 8.0), 100 mM NaCl, 1 M EDTA, and 1 mM DTT. To this mixture, 1 mM PMSF and 0.2 to 50 mg/ml lysozyme were added and incubated at 4 °C for 30 min. Triton X-100 was then added at a final concentration of 0.2%. After sonication, the mixture was centrifuged at 17,000*g* and 4 °C for 5 min and then washed three times with buffer B containing a final concentration of 50 mM Tris–HCl (pH 8.0), 100 mM NaCl, 1 M EDTA, 1 mM DTT, and 1% Triton X-100. The mixture was then spun down at 17,000*g* for 5 min at 4 °C and treated with 25 μg/ml DNase I incubation buffer (40 mM Tris–HCl [pH 8], 10 mM NaCl, 6 mM MgCl_2_, and 1 mM CaCl_2_) and one tablet of the Protease Inhibitor Cocktail cOmplete EDTA free (Merck). The mixture was rotated at 20 to 25 °C for 1 h. The mixture was then centrifuged at 17,000*g* and 4 °C for 5 min. Buffer A was added to the bacterial pellet and sonicated. The bacteria were centrifuged at 17,000*g* and 4 °C for 5 min. After that, 16 ml 8 M urea solution containing 8 M urea, 25 mM 2-(*N*-morpholino)ethanesulfonic acid, 10 mM EDTA pH 8.0, 0.1 mM DTT, and 0.5 mM PMSF was added to the bacterial culture, rotated at 20 to 25 °C for 2 to 3 h, and centrifuged at 17,000*g* and 20 °C for 20 min. Thereafter, the supernatant was harvested to collect the inclusion bodies.

### Refolding of pMHC and biotinylation

First, 200 ml of refolding buffer was prepared using 100 mM Tris–HCl (pH 8.0), 400 mM L-arginine-HCl, 2 mM EDTA (pH 8.0), 0.5 mM oxidized glutathione, and 5 mM reduced glutathione. To this buffer, 2 ml of 100 mM PMSF was added, followed by 100 μl pepstatin dissolved in 2 mg/ml dimethyl sulfoxide, 100 μl leupeptin dissolved in 2 mg/ml distilled water, and 500 μl 20 mg/ml peptide. Next, 4.4 mg human β-2-microglobulin in 500 μl guanidine solution containing 3 M guanidine-HCl, 10 mM sodium acetate, and 10 mM NaEDTA were added along with 6.2 mg HLA-A∗24:02 in 500 μl guanidine solution to the mixture and slowly stirred at 4 °C for 24 h. This step was repeated three times. The refolding buffer/protein was then poured into a dialysis tube and dialyzed in 1.8 L distilled water at 4 °C for 24 h with stirring. The protein was then purified with Superdex 200 HR 100/300 on an AKTA Purifier (GE HealthCare) and biotinylated overnight. Biotinylated refolded protein was analyzed using Superdex 200 HR 100/300 on an AKTA Purifier. Thereafter, biotinylated protein was concentrated, stored at −80 °C, and thawed for use.

### Affinity enhancement

Based on a previous study (18), we introduced mutations into the WT98 DNA using oligonucleotides (Table S4) and PCR. We generated four variants (98A, 98G, 98J, and 98B) by replacing three amino acids, including serine and threonine, with arginine in the framework region 3 of the light chain variable region. These variants were used in this study.

### Vector construction and preparation of retroviral solution

Each scFv domain, WT163, WT98, hinge domain of the constant region of the lambda light chain, CD28 transmembrane domain, intracellular signaling domain of CD3ζ, and glucocorticoid-induced tumor necrosis factor receptor family-related protein as well as the variants 98A, 98G, 98J, and 98B were inserted into a pMS3 retroviral vector (Takara Bio). Packaging cell lines, namely Platinum-A cells (Cell Biolabs), were seeded in Dulbecco’s modified eagle medium (Wako) supplemented with 10% fetal calf serum (Gibco), 100 U/ml penicillin, and 100 μg/ml streptomycin in 6-well plates. On the following day, the cells of each well that reached 60 to 80% confluency were transfected with 3 μg pMS3 and 0.2 μg pVSV with lipofectamine LTX (Invitrogen) using Opti-MEM (Thermo Fisher Scientific) according to the manufacturer’s instructions. The pVSV vector was kindly provided by Dr Emi of the Fujita Health University, Japan. The cell supernatant was harvested after 48 h by filtering it through a 0.45-μm mixed cellulose ester membrane filter.

### Generation of CAR-T cells

PBMCs were isolated from the healthy donor peripheral blood. According to the guidelines of the declaration of Helsinki adopted by the World Medical Association, all PBMCs were obtained after written informed consent. The experimental protocol was approved by the Ethics Committee of Mie University School of Medicine (approval number: 3264). PBMCs were activated on a plate coated with anti-human CD3 antibody (5 μg/ml; OKT3 Janssen Pharmaceutica) and RetroNectin (25 μg/ml; Takara Bio). The cells were cultured in GT-T551 (Takara Bio) supplemented with 200 IU/ml human recombinant IL-2 and 0.6% autologous human plasma. After 3 days of stimulation, retroviral supernatants were loaded onto a RetroNectin-coated plate and centrifuged at 2000*g* and 32 °C for 2 h. After coating the retrovirus onto the RetroNectin-coated plate, PBMCs were added to the plate by centrifugation at 1000*g* and 32 °C for 10 min. The following day, primary-transduced PBMCs were used to repeat transduction. The transduced PBMCs were then expanded for an additional 6 days before being used for experiments on days 10 to 16.

### Peptide pulsing

TAP-deficient T2 cells transduced with HLA-A∗24:02 gene (T2A24) were washed with serum-free RPMI 1640 and incubated at 1 × 10^6^ cells/ml in serum-free RPMI 1640, including the peptides, at the indicated concentrations at 37 °C for 2 to 3 h.

### Enzyme-linked immunosorbent assay

Effector cells (5 × 10^4^ cells) were coincubated with peptide-pulsed T2A24 cells (5 × 10^4^ cells) in a 96-well U plate. After 24 h, the culture medium was centrifuged at 500*g* for 5 min. ELISA was performed using a human granzyme B (MABTECH 3486-1H-6) ELISA kit according to the manufacturer’s protocol. The plates were analyzed using a plate reader (Bio-Rad) at wavelengths of 450 to 550 nm.

### Lactate dehydrogenase-based cytotoxicity assay

T2A24 cells (3 × 10^4^ cells) were pulsed with the indicated peptides and coincubated with effector cells (9 × 10^4^ cells) in U-bottom 96-well plates for 4 h. A nonradioactive cytotoxicity assay, CytoTox96 (Promega), was performed according to the manufacturer’s instructions. Then, 50 μl of the supernatant was transferred to an ELISA plate containing 50 μl CytoTox 96 Reagent. After incubation at 20 to 25 °C for 30 min, stop solution was added to each well. The absorbance was immediately measured at a wavelength of 490 nm. The lactate dehydrogenase release was measured according to the manufacturer’s instructions.

### Real-time PCR

Total RNA was isolated from cultured cell lines according to the manufacturer’s protocol (QIAGEN). The QuantiTect Reverse Transcription Kit (QIAGEN) was used to reverse-transcribe the total RNA. The relative expression of PRAME was normalized to that of GAPDH for each sample. Applied Biosystems provided the TaqMan probes (PRAME:HS01022301m1; GAPDH:4325792). The samples were quantified using a StepOnePlus Real-Time PCR System (Applied Biosystems) and analyzed using StepOne Software v2.3 (https://www.thermofisher.com/jp/ja/home/technical-resources/software-downloads/StepOne-and-StepOnePlus-Real-Time-PCR-System.html). The quantification range was 5–5 × 10^8^ copies, including PRAME-plasmid DNA and GAPDH-plasmid DNA. The thermal cycling conditions were as follows: one cycle at 50 °C for 2 min, one cycle at 95 °C for 10 min, and 40 cycles at 95 °C for 15 s, and at 60 °C for 1 min.

### Flow cytometry

The transduction efficiency of PBMCs was evaluated using a 1:300 rabbit anti-lambda antibody (MBL), 1:200 anti-rabbit IgG polyclonal Ab conjugated with Alexa488 (Invitrogen), 1:200 anti-CD4 PerCPcy5.5 (RPA-T4; BioLegend), and 1:200 anti-CD8 PEcy7 (RPA-T8, BioLegend) 6 or 7 days after primary viral transduction. To measure CAR-T cell production, we cocultured CAR-T cells with T2A24 cells pulsed with 10 μM peptides of PRAME_p301_ and CMV_pp65_ at 37 °C for 5 h in the presence of GolgiStop (BD Bioscience) and 1:100 anti-CD107a APC (H4A3, BioLegend). The CAR-T cells were then stained with 1:300 anti-lambda antibody (MBL), 1:200 anti-rabbit IgG Alexa488 (Invitrogen), 1:200 anti-CD4 PerCPcy5.5 (RPA-T4, BioLegend), and 1:200 anti-CD8 PEcy7 (RPA-T8, BioLegend). To assess the expression of HLA-A∗24:02 on the cell surface, the cell lines were stained with HLA-A24,23 bulk monoclonal antibodies (One Lambda, Thermo Fisher Scientific) at a 1:200 dilution at 20 to 25 °C for 30 min. This was followed by incubation with an anti-mouse-IgG polyclonal antibody conjugated with Alexa488 (Invitrogen) at a 1:200 dilution at 20 to 25 °C for 30 min. To detect the PRAME_p301_/HLA-A∗24:02 complex on the cell surface, soluble WT98 was reacted with the tumor cells at 20 to 25 °C for 30 min. Subsequently, the cells were stained with a 1:300 dilution of rabbit anti-lambda antibody (MBL) and incubated with a 1:200 dilution of R-phycoerythrin-conjugated anti-rabbit IgG polyclonal antibody (Invitrogen). Flow cytometry analyses were performed using LSRFortessa (BD Biosciences), and the data were analyzed using FlowJo v.10 (Tre Star, Ashland, https://www.flowjo.com/solutions/flowjo/downloads).

### Real-time killing assays using the xCELLigence system

NW-38, NW-38A24, SK-MEL-124, and SK-MEL-128 cells were cultured on xCELLigence E plates (5469830001, Agilent) at a density of 1 × 10^4^ cells/well. After 21 to 23 h, CAR-T cells were added at E:T ratios of 5:1, 1.5:1, 0.5:1, and 0.15:1. Cellular impedance increases as adherent cells proliferate; however, as effector cells kill adherent cells, they separate from the plate and cellular impedance decreases. Cellular impedance was recorded at 2-min intervals for 50 h after the addition of effector cells.

### Calculation of killing rates

To calculate the killing rates as a rate constant of CAR-T cells, xCELLigence data over 24 h were obtained with a constraint of constant values Y0 = 1 and Plateau = 0 to fit the one-phase exponential decay equation, and rate constants were established using the 95% confidence interval. When the 95% confidence interval for the rate constant could not be determined, it was defined as 0.

### Statistical analysis

The relationship between the two variables was determined using simple linear regression of the log-transformed axes. To calculate the killing rates from the xCELLigence raw data, a one-phase exponential decay model was used for curve fitting. Figure 1*D* was analyzed using two-way ANOVA with Tukey’s test. Fig. S5 was analyzed using one-way ANOVA with Dunnett’s test in comparison with WT98. Statistical significance for multiple comparisons in other figures was determined using one-way ANOVA with Tukey’s test. Statistical analyses were performed using GraphPad Prism 9 (https://www.graphpad.com).

## Data availability

All data from this study are contained in this manuscript and the supporting information. Nucleotide sequence data reported are available in the DNA DataBank of Japan database under the accession numbers LC820886 and LC820887.

## Supporting information

This article contains supporting information.

## Conflicts of interest

Hiroyuki Hiratsuka and Yasushi Akahori have filed provisional patent applications for the sequence data of WT98 (WO 2022124282-A/1–WO 2022124282-A/8) and WT163 (JP2024–115711). The late Hiroshi Shiku was involved in the patent application for WT98. Part of this research was funded by a cooperative research grant from Sysmex Corporation. Shingo Maeta and Yuriko Egashira are affiliated with Sysmex Corporation.
