# Supplementary material for: Fast on-rates of chimeric antigen receptors enhance the sensitivity to peptide MHC via antigen rebinding
Source: J Biol Chem. 2024 Aug 8;300(9):107651. doi: 10.1016/j.jbc.2024.107651 (PMC11407991; doi:10.1016/j.jbc.2024.107651)
Supplement: Supplementary Figures [file mmc1.docx]

**Supporting Information**

**Fast on-rates of chimeric antigen receptors enhance the sensitivity to peptide MHC via antigen rebinding**

Hiroyuki Hiratsuka^a,^*, Yasushi Akahori^a,^*, Shingo Maeta^b^, Yuriko Egashira^b^, and Hiroshi Shiku^a,c,†^

**This file includes:**

Figures S1–S7


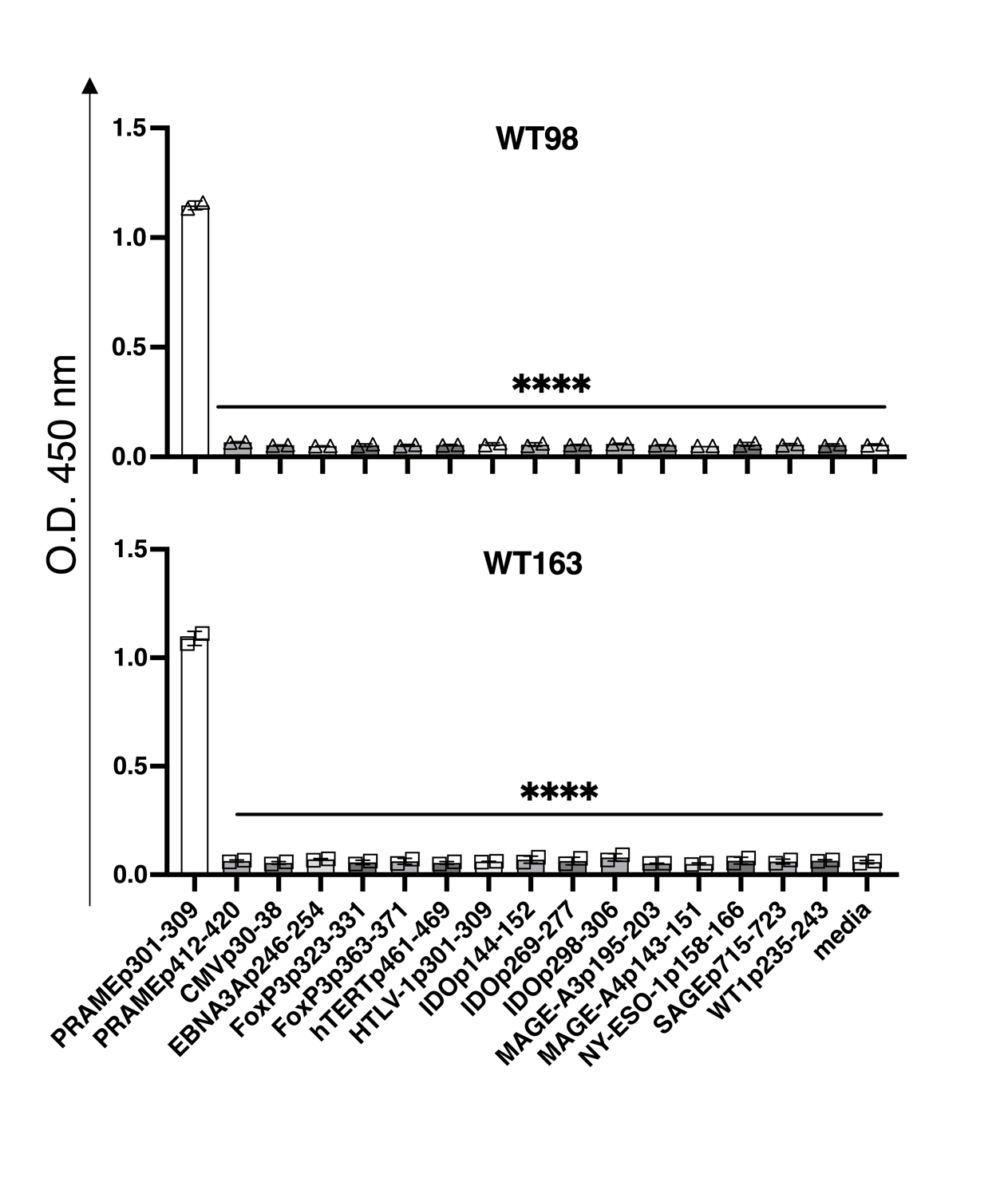


**Figure S1.** Isolation of clones WT98 and WT163. WT98 and WT163 supernatants of 2 × YT medium, including ampicillin and IPTG, were reacted with biotin-conjugated pMHCs immobilized on neutravidin-coated plates and detected by anti-cp3 antibody and HRP-conjugated anti-mouse IgG using ELISA. Data are presented as the mean ± SDs of two independent experiments. **** *p <* 0.0001 was observed using one-way ANOVA with Tukey’s test performed to compare PRAMEp301-309 and other peptides.

**
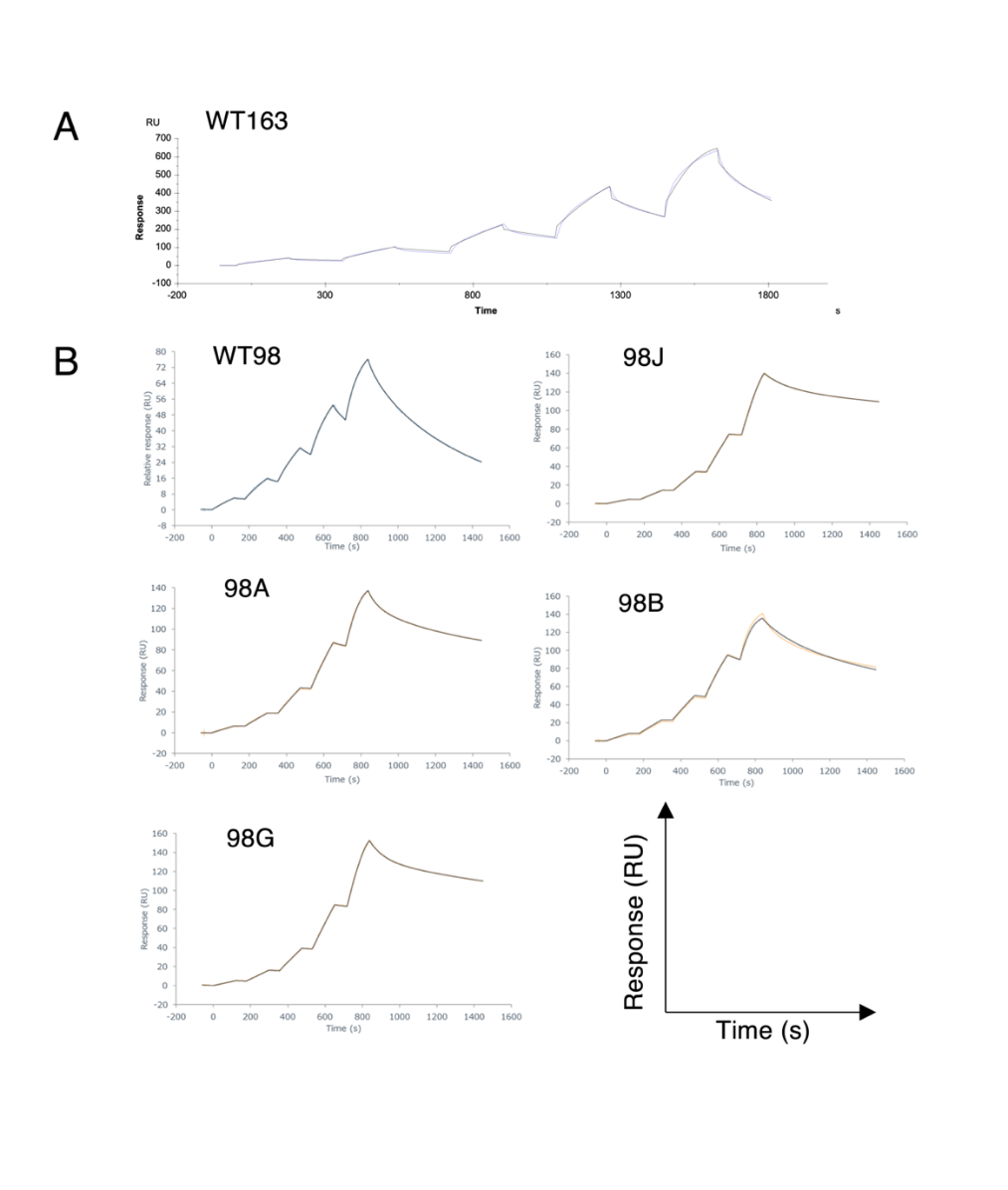
**

**Figure S2.** Surface plasmon resonance (SPR) measurements of WT163, WT98, and WT98 mutants. (A) SPR experiments of WT163 protein binding to biotinylated PRAME_p301-309_/HLA-A*24:02 monomers at 25 °C. pMHC was immobilized on a biotin capture sensor chip and WT163 proteins were added to the flow cell of the chip and allowed to flow. The association and dissociation phase data were simultaneously fitted using a 1:1 model. (B) SPR measurements of WT98 and mutant protein binding to biotinylated PRAME_p301-309_/HLA-A*24:02 monomers at 25 °C. Biotinylated pMHC was immobilized on a biotin capture sensor chip, and WT98 and mutant proteins were added to the flow cell of the chip and allowed to flow. The association and dissociation phase data were simultaneously fitted to the bivalent binding curve.


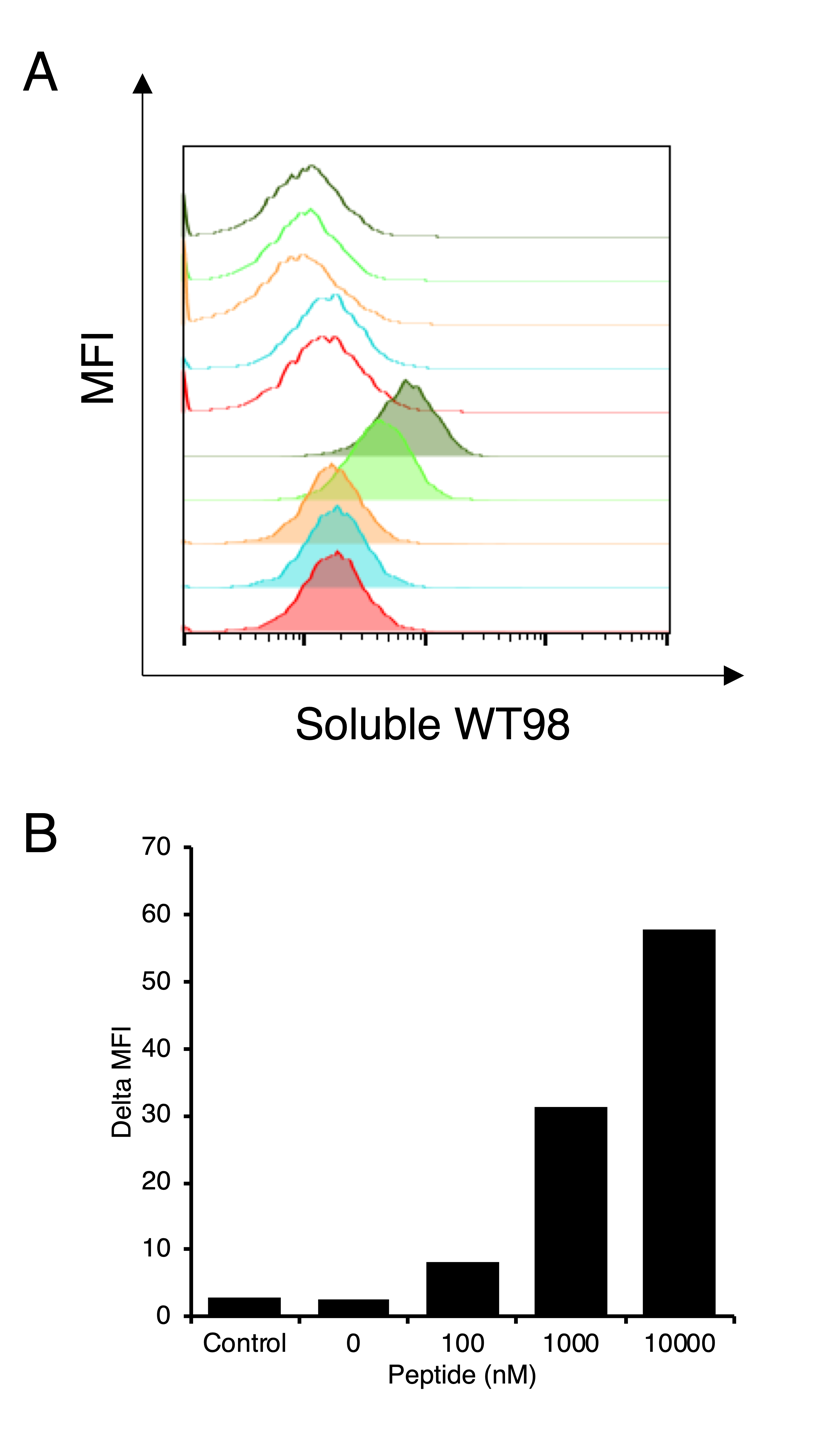


**Figure S3.** pMHC detection using soluble WT98 against peptide-pulsed HLA-A*24:02^+^ lymphoblastoid cell line (LCL). (A) LCL cells were pulsed with the indicated peptides and reacted with soluble WT98. pMHC was then detected by staining these cells with 1:300 rabbit anti-lambda antibody (MBL, Tokyo, Japan) and 1:200 anti-rabbit IgG polyclonal antibody conjugated with PE (Invitrogen, Waltham, MA, USA). Thereafter, flow cytometry was performed. (B) Histogram of (A) Delta MFI = MFI (filled color) – without soluble WT98 MFI (not filled color).


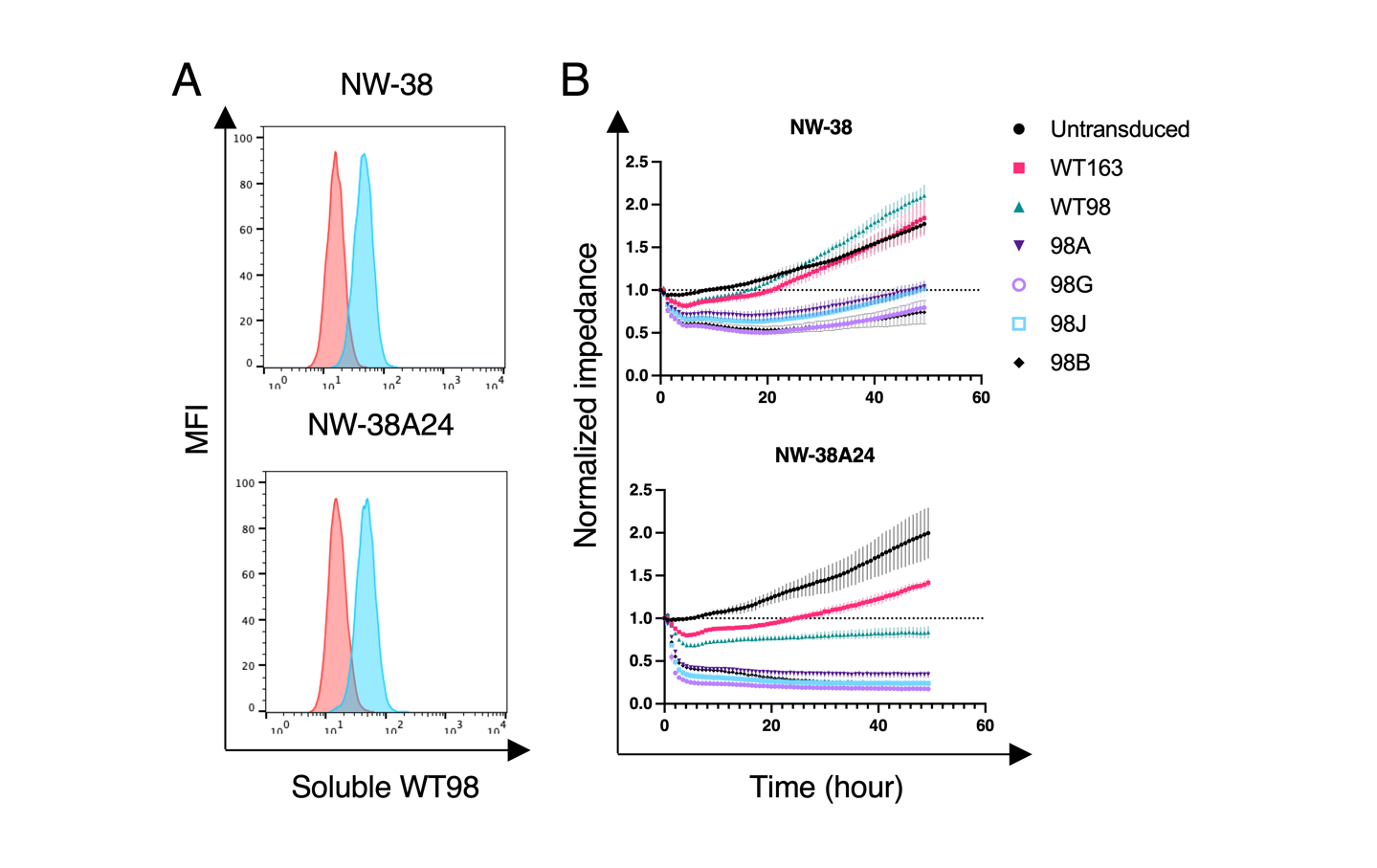


**Figure S4.** CAR-T cells induced specific killing of HLA-A*24:02-transduced NW-38 cells but not untransduced NW-38 cells. (A) pMHC detection using soluble WT98 for PRAME^+^/HLA-A*24:02^-^ NW-38 and NW-38A24 cells compared with that without soluble WT98. (B) Specific cytotoxicity was measured using the xCELLigence system at an E:T ratio of 5:1. Data are representative of two independent experiments and are presented as the mean ± SDs of triplicates.


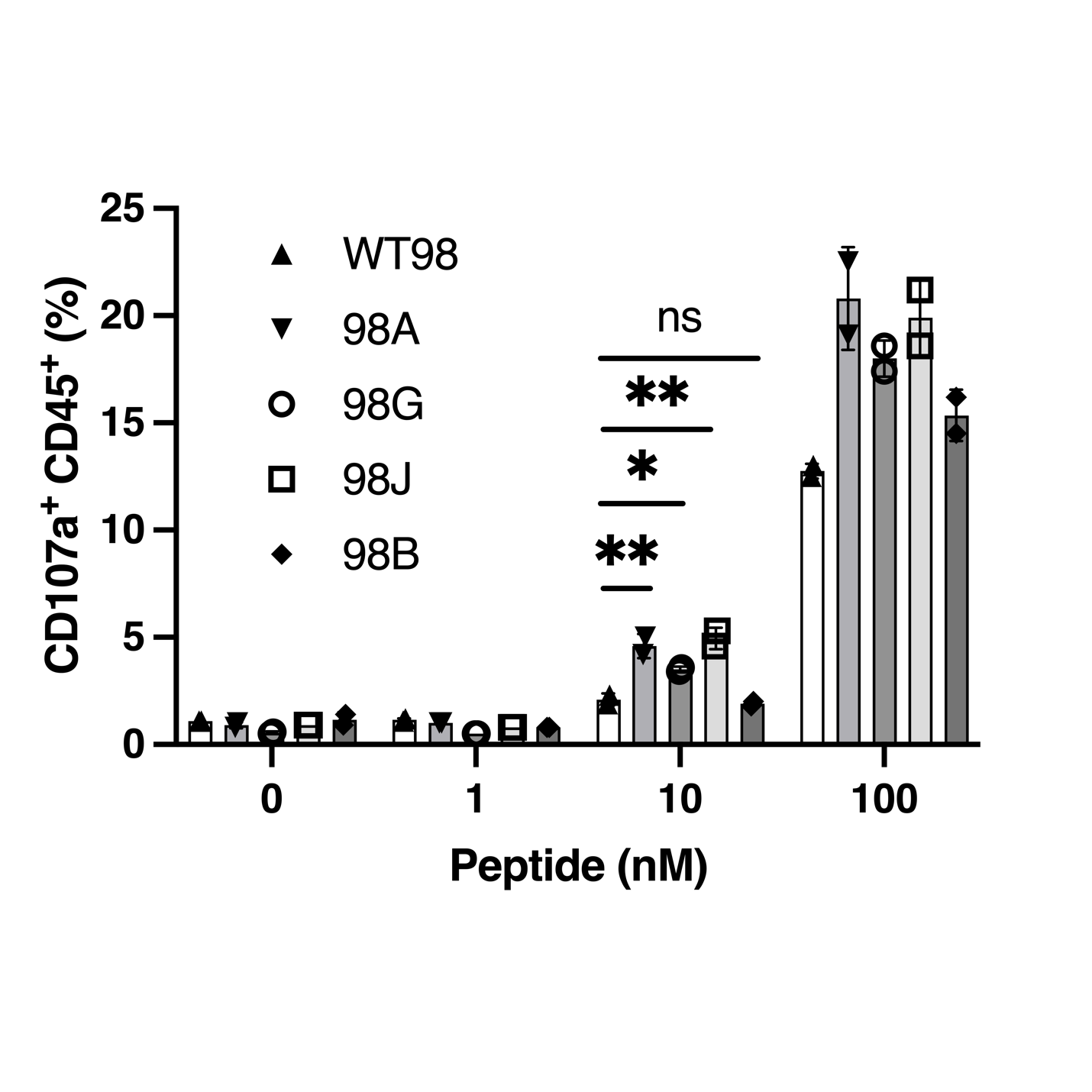


**Figure S5.**  Degranulation assay of CAR-T cells over a short period. CAR-T cells (5 × 10^4^ cells) were incubated with peptide-pulsed T2A24 cells (5 × 10^4^ cells) and cultured with 1:100 anti-human CD107a-APC. After 1 h, CAR-T cells were stained with 1:125 anti-human CD45-V450 (clone HI30) for 15 min. Thereafter, flow cytometry was performed. Data are presented as the mean ± SDs of duplicates. ** *p* < 0.01, * *p* < 0.05; ns, not significant results of one-way ANOVA with Dunnett’s test comparing the clones with WT98.

**
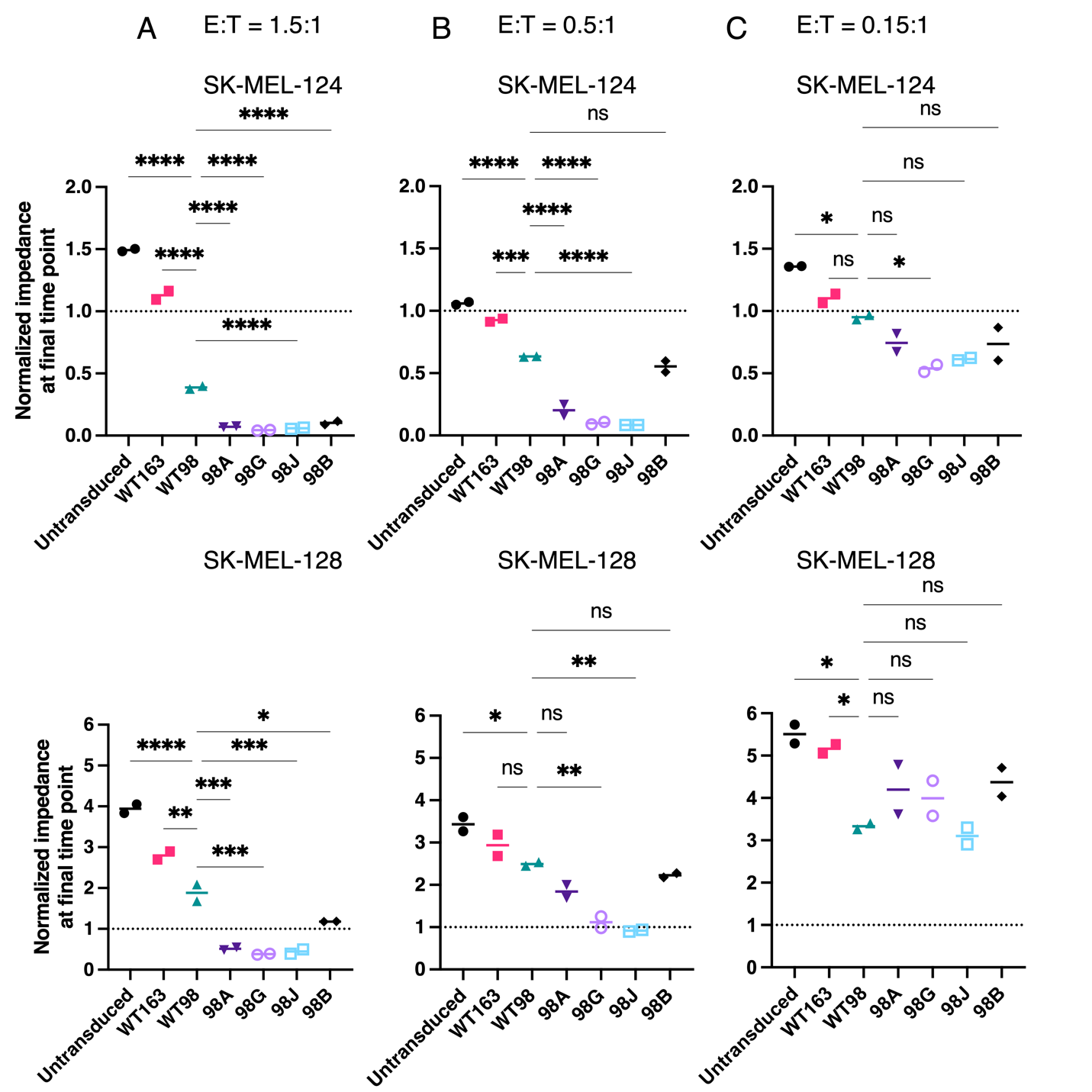
**

**Figure S6.** Threshold of CAR-T cell-killing capacity. (A) CAR-T cells were cocultured at E:T ratios of (A) 1.5:1, (B) 0.5:1, and (C) 0.15:1 with SK-MEL-124 or SK-MEL-128; untransduced T cells were cocultured as a control. Data were recorded as the average impedance over time, which was normalized to the time point at which the effector cells were added. These dot plots show the final time point of the normalized impedance. Data are representative of two independent experiments and are presented as mean ± SDs of duplicates. **** *p <* 0.0001, *** *p <* 0.001, ** *p <* 0.01, * *p <* 0.05; ns, not significant results of one-way ANOVA with Tukey’s test.

**
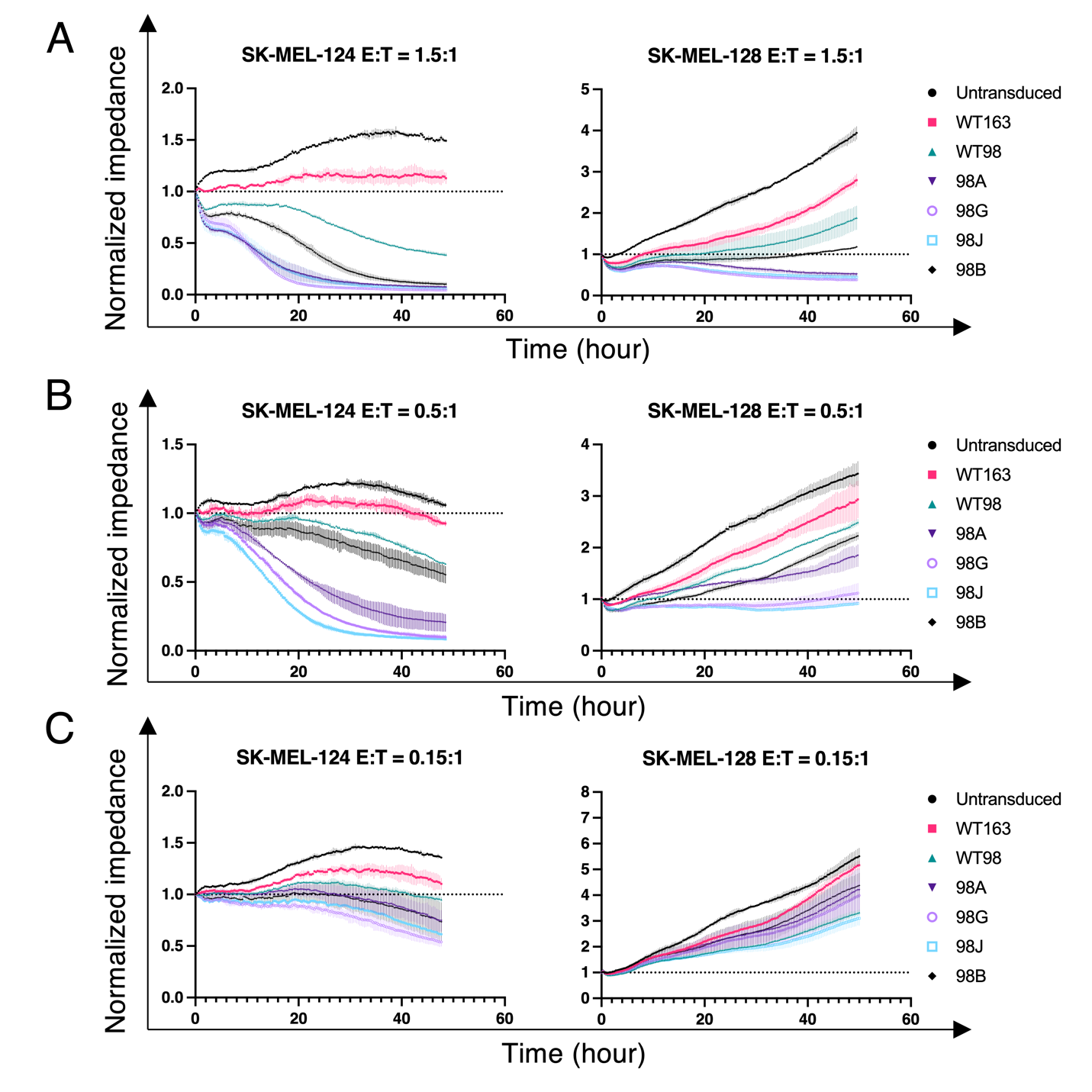
**

**Figure S7.** Representative data from the xCELLigence system are shown in Figure S6.
