# Supplementary material for: Fast on-rates of chimeric antigen receptors enhance the sensitivity to peptide MHC via antigen rebinding
Source: J Biol Chem. 2024 Aug 8;300(9):107651. doi: 10.1016/j.jbc.2024.107651 (PMC11407991; doi:10.1016/j.jbc.2024.107651)
Supplement: Supplementary Tables [file mmc2.docx]

**Supporting Information**

**Fast on-rates of chimeric antigen receptors enhance the sensitivity to peptide MHC via antigen rebinding**

Hiroyuki Hiratsuka^a,^*, Yasushi Akahori^a,^*, Shingo Maeta^b^, Yuriko Egashira^b^, and Hiroshi Shiku^a,c,†^

**This file includes:**

Tables S1–S4

**Table S1**. WT163 and WT98 sequence data used for CAR-T cells.

| Clone | Signal sequence | VH | Single chain | VL | C lambda |
| --- | --- | --- | --- | --- | --- |
| **WT163** | MKHLWFFLLLVAAPRWVLSQVQL | **EVQLVESGGGLVQPGGSLRLSCAASGFTFSSYAMSWVRQAPGKGLEWVSAISGGGGSTYYADSVKGRFTISRDNSKNTLYLQMNSLRAEDTAVYYCAKWRSAYGSTNYYYYGMDVWGQGTTVTVSR** | GGGGSGGGGSGGGGSMA | **SSELTQDPAVSVALGQTVTITCQGDSLRKYYASWYQQKPGQAPVFVFYGKNNRPSGIPDRFSGSSSGDTASLTITGAQAEDEADYYCNSRDRGSNQVVFGGPTRLTVL** | GQPKAAPSVTLFPPSSEELQANRAALVCLISDFHPGAVTVAWKADSSPVKAGVETTTPSKQSNNKYAASSYLSLTPEQWKSHRSYSCQVTHEGSTVEKTVAPTECSARQ |
| **WT98** | MKHLWFFLLLVAAPRWVLSQVQL | **QVQLVQSGAEVKKPGSSVKVSCKASGGTFSSYAISWVRQAPGQGLEWMGGIIPIFGTANYAQKFQGRVTITADESTGTAYMELSSLRSEDTAVYYCARHHSNYYYYGMDVWGQGTTVTVSR** | GGGGSGGGGSGGGGSMA | **SYELTQPLSVSVALGQTARITCGGNNIGSKNVHWYQQKPGQAPVLVIYRDSNRPSGIPERFSGSNSGNTATLTISRAQAGDEADYYCQVWDSSHVFGTGTKVTVL** | GQPKANPTVTLFPPSSEELQANKATLVCLISDFYPGAVTVAWKADGSPVKAGVETTKPSKQSNNKYAASSYLSLTPEQWKSHRSYSCQVTHEGSTVEKTVAPTECSARQ |

**Table S2**. Synthetic peptide sequences for alanine scanning. Inhibitory concentration 50 (IC_50_) and percentile rank of the peptides for HLA-A*24:02 were predicted using NetMHCPan4.0.

| Name | Sequence | IC_50_ (nM) | Percentile rank |
| --- | --- | --- | --- |
| PRAME_p301_ | LYVDSLFFL | 59.2 | 0.1 |
| L1A | AYVDSLFFL | 59.2 | 0.1 |
| Y2A | LAVDSLFFL | 12,500.8 | 5.8 |
| V3A | LYADSLFFL | 43.7 | 0.08 |
| D4A | LYVASLFFL | 39.8 | 0.07 |
| S5A | LYVDALFFL | 73.7 | 0.12 |
| L6A | LYVDSAFFL | 85.7 | 0.13 |
| F7A | LYVDSLAFL | 178.8 | 0.23 |
| F8A | LYVDSLFAL | 129.6 | 0.19 |
| L9A | LYVDSLFFA | 3,565.3 | 2.1 |
| CMV_pp65_ | QYDPVAALF | 66.55 | 0.11 |

**Table S3**. Predicted wild-type peptides derived from the human genome for CAR 98B using MOTIF Search and NetMHCPan4.0

| Number | Name | Amino acid sequence | IC_50_ | Percentile rank |
| --- | --- | --- | --- | --- |
| - | PRAME | LYVDSLFFL | 59.2 | 0.1 |
| 1 | CYP4F8 | RYKDFLYFL | 17.9 | 0.03 |
| 2 | EBP | IYGDVLYFL | 19.9 | 0.04 |
| 3 | USP25 | SYIDSLLFL | 47.9 | 0.08 |
| 4 | RSBN1 | AYMDELSFL | 80 | 0.13 |
| 5 | STON1 | SYHDFLDFL | 116.1 | 0.18 |
| 6 | STON1-GTF2A1L | SYHDFLDFL | 116.1 | 0.18 |
| 7 | FARP1 | AYQDTLEFL | 146.6 | 0.2 |
| 8 | PSMD13 | YYKDALRFL | 168.2 | 0.22 |
| 9 | CSF1R | CYGDLLNFL | 178.9 | 0.23 |
| 10 | KIT | CYGDLLNFL | 178.9 | 0.23 |
| 11 | PRPF40A | IYEDVLFFL | 191.7 | 0.25 |
| 12 | FARP2 | PYQDTLEFL | 241.6 | 0.3 |
| 13 | SHH | LYSDFLTFL | 249.7 | 0.31 |
| 14 | RTCB | SYNDELQFL | 263 | 0.33 |
| 15 | PRPF40B | VYDDVLFFL | 369.1 | 0.41 |
| 16 | PNPLA5 | GYLDALRFL | 433.7 | 0.44 |
| 17 | CATSPERB | LYYDHLGFL | 539.7 | 0.53 |
| 18 | OR8U3 | FYCDDLPFL | 680 | 0.62 |
| 19 | PTH2R | NYSDCLRFL | 756.4 | 0.68 |
| 20 | PNPLA2 | GYRDGLRFL | 3415 | 2 |

**Table S4.** Oligonucleotides used to generate mutants of WT98 displayed in this study using affinity engineering methods.

| Name | Sense | Sequence (5ʹ to 3ʹ) | Mutant position |
| --- | --- | --- | --- |
|  |  |  | Amino acids |
| WT98 | - | - | **S**63 **S**65 **S**67 **T**70 **T**72 |
|  |  |  | PERF**S**G**S**N**S**GN**T**A**T**LTI |
| 98A | Forward | CATTCTCTGGCCGAAACCGAGGGAACCGAGCCACCCTG | S63 **R**65 **R**67 **R**70 T72 |
|  | Reverse | CAGGGTGGCTCGGTTCCCTCGGTTTCGGCCAGAGAATC | FSG**R**N**R**GN**R**ATL |
| 98G | Forward | GCGATTCCGAGGCTCCAACCGAGGGAACACGGCCCGACTGACCATC | **R**63 S65 **R**67 T70 **R**72 |
|  | Reverse | GATGGTCAGTCGGGCCGTGTTCCCTCGGTTGGAGCCTCGGAATCGC | RF**R**GSN**R**GNTA**R**LTI |
| 98J | Forward | AGCGATTCCGAGGCCGAAACCGAGGGAACAC | **R**63 **R**65 **R**67 T70 T72 |
|  | Reverse | GTGTTCCCTCGGTTTCGGCCTCGGAATCGCT | RF**R**G**R**N**R**GN |
| 98B | Forward | CCCTGAGCGATTCCGAGGCCGAAACTCGGGGAACCGAGCCAC | **R**63 **R**65 S67 **R**70 T72 |
|  | Reverse | AGGGTGGCTCGGTTCCCCGAGTTTCGGCCTCGGAATC | PERF**R**G**R**NSGN**R**A |
